# Habitual low carbohydrate high fat diet compared with omnivorous, vegan, and vegetarian diets

**DOI:** 10.3389/fnut.2023.1106153

**Published:** 2023-04-13

**Authors:** Nives Bogataj Jontez, Saša Kenig, Karin Šik Novak, Ana Petelin, Zala Jenko Pražnikar, Nina Mohorko

**Affiliations:** Faculty of Health Sciences, University of Primorska, Izola, Slovenia

**Keywords:** low carbohydrate high fat diet, vegan diet, vegetarian diet, omnivorous diet, serum cholesterol, diet quality, saturated fatty acid, monounsaturated fatty acid

## Abstract

**Background:**

Dietary patterns which exclude whole food groups, such as vegetarian, vegan and low carbohydrate high fat diet (LCHF), are increasingly popular in general public. When carefully planned, all these diets have some known benefits for health, but concerns are also raised in particular for LCHF. The quality of LCHF diet which individuals follow in real life without supervision is not known.

**Methods:**

One hundred thirty healthy individuals with stable body mass following LCHF, vegan, vegetarian and omnivorous diet for at least six months, were compared in a cross-sectional study. Diet was analyzed through 3-day food records and FFQ, anthropometric measurements were performed and serum metabolic biomarkers determined from fasting blood.

**Results:**

Participants on LCHF diet had the intakes of micronutrients comparable to other groups, while the intakes of macronutrients differed in line with the definition of each diet. The intakes of saturated fats, cholesterol and animal proteins were significantly higher and the intakes of sugars and dietary fibers were lower compared to other groups. Healthy eating index 2015 in this group was the lowest. There were no differences in the levels of glucose, triacylglycerols and CRP among groups. Total and LDL cholesterol levels were significantly higher in LCHF group, in particular in participants with higher ketogenic ratio. Fatty acids intakes and intakes of cholesterol, dietary fibers and animal proteins explained 40% of variance in total cholesterol level, with saturated fatty acids being the strongest positive predictor and monounsaturated fatty acids a negative predictor.

**Conclusion:**

None of the self-advised diets provided all the necessary nutrients in optimal levels. Due to the detected increased levels of serum cholesterols, selection of healthy fat sources, higher intake of dietary fibers and partial replacing of animal sources with plant sources of foods should be recommended to the individuals selecting LCFH dietary pattern.

**Clinical Trial Registration**: ClinicalTrials.gov, identifier NCT04347213.

## 1. Introduction

Traditionally the majority of people in Western countries and also in Slovenia were omnivores. National guidelines for healthy nutrition are thus based on omnivorous diet (1). In the last decades the popularity of different dietary patterns such as vegetarian, vegan and low carbohydrate high fat (LCHF) is rising, according to lay publications, social media and cross sectional surveys (2, 3). The motivators for choosing such a dietary pattern may be ethical and environmental issues, weight loss or improving fitness, but the main motivation is improving health (4, 5). All mentioned dietary patterns are in fact advertised in public as health beneficial and safe for long term practicing, but for LCHF, most scientific evidence originates from intervention studies, mostly with ketogenic diet and rarely longer than 15 weeks. On the other hand, little is known of the long-term effects in healthy adults, especially when the dietary choices in diets that omit whole food groups are made without proper counseling. Although nation-representative scans, performed under the umbrella of EFSA, investigate the connections between habitual dietary pattern and health (6), LCHF, vegetarian and vegan dietary patterns are rare, so the data of individuals following those dietary patterns cannot be extracted from such studies and no data is available about them.

In order for a diet to be considered low carbohydrate, carbohydrate intake is limited to 130 g daily or <26% of daily energy intake (EI) for a 2,000 kcal/day diet (7), and thus this pattern excludes or strongly limits the intake of starchy foods, legumes, sugars and fruits (8). To compensate for the described omissions, subjects following such a dietary pattern consume higher amounts of concentrated fats, meat, poultry, fish, eggs and cheese as well as red and processed meat (9). The majority of energy therefore derives from fat, making their fat intake higher than in dietary patterns that do not restrict carbohydrates. Some LCHF diets restrict carbohydrate intake to the extent to promote ketogenesis, making them ketogenic LCHF, while the others allow enough carbohydrate that the ketogenesis does not take place, making them nonketogenic LCHF (10). For the purpose of this article, we will call LCHF a dietary pattern that limits carbohydrates to 26% EI or less and has fat intakes more than 50% EI which is 167% of the recommended fat intake of 30% EI (11). LCHF is also the term with which such a dietary pattern is presented in lay literature in Slovenia and under which the persons that follow it identify themselves. Vegetarian and vegan dietary patterns also exclude whole food groups, as vegetarians eliminate meat, poultry and often fish, and vegans in addition reject all animal products such as dairy and eggs, and other products from animal origin, such as honey (12). The predominance of different food groups in the diet reflects in an altered intake of macronutrients which have differential effects on metabolism and health. LCHF is defined by high fat intake, and there is typically low intake of dietary fibers, while vegetarians and especially vegans usually have higher intakes of carbohydrate, omega-6 fatty acids and dietary fibers, but lower intake of protein, saturated fatty acids (SFA) and long chain omega-3 fatty acids (13). All restrictive dietary patterns also carry the risk of micronutrient deficiencies. Thus, restricting carbohydrate rich foods in LCHF may lead to low intake of thiamin, folate, niacin, riboflavin, vitamins A, C, and E, pyridoxine, calcium, magnesium, iron, potassium, selenium, and zinc (14, 15). Without supplementation, deficiencies in vitamin K, linolenic acid and water-soluble vitamins, excluding vitamin B_12_, are common (9). Vegetarians and more often vegans may be vitamin B_12_ deficient, because vitamin B_12_ is mainly found in animal source foods (2). More severe vitamin D deficiencies are also found among vegans, compared with other dietary patterns (16).

As with all major lifestyle changes, health benefits were found to be among the main motivators also for people who have switched to LCHF (4). Indeed, metabolic disturbances that may be improved with LCHF diet [such as glucose metabolism, triacylglicerols levels and blood pressure (17)] are often the cause of various diseases, especially chronic noncommunicable diseases. Diet quality and intake of bioactive compounds are independent risk factors for non-communicable diseases and all-cause mortality (18, 19). Certain promotors of vegan, vegetarian or LCHF dietary patterns often consider only their dietary pattern as healthy and all the other dietary patterns wrong, creating myths in the public. Whether any of these patterns can be considered healthy when it is self-advised and used over a long term, may depend on the choice and quality of the food chosen from allowed food groups by the individual and observed/measured parameter, and remains controversial, especially for LCHF, for which epidemiological research is scarce. A high fat intake, high in SFA, is a well-established risk factor for high serum triacylglycerols and cholesterol levels. However, in majority of population high fat and particularly high SFA intake is associated with high sugar intake as part of a Western diet. Metabolic millieu of individuals on LCHF dietary pattern is different to the ones on Western diet due to low carbohydrate intake (20, 21). Short-term studies have been inconclusive about the effects of LCHF dietary pattern on serum cholesterol and triacylglycerols levels (9, 20, 22, 23). LCHF dietary pattern in overweight and obese subjects with weight loss has been associated with improved fat and glucose metabolism on short-term (20, 24), but long-term effects without energy restriction are unknown. On the upside, adherence to LCHF dietary pattern for a short period was associated with increased insulin sensitivity, better glucose regulation and lower risk for metabolic syndrome (9). Contrary to the LCHF diet, vegetarians and vegans consume less dietary fats, in particular SFA, and also exhibit lower total serum and LDL cholesterol levels than omnivores (2). In fact, vegetarian dietary pattern was associated with cardiovascular disease prevention (25). Energy intake is an important parameter as well, as it is important to maintain body mass. Lower body mass index (BMI) was determined among vegetarians and vegans, which was associated with a lower risks of developing obesity, metabolic syndrome and type 2 diabetes mellitus (25).

It has been established that vegetarians and vegans have lower mortality rates from chronic noncommunicable diseases (13), but this result may be partly due to an overall healthier lifestyle. For instance, they often avoid smoking and drinking alcohol, have lower BMI and higher levels of physical activity than omnivores (2). However, diets low in carbohydrate are also associated with improved health parameters (20). Ketone bodies, which are synthesized when the usage of fatty acids is elevated, have been linked to the alleviation of several age-related diseases and to longevity [reviewed in (26)]. It is known that with careful planning, one can achieve the recommended levels of all nutrients regardless of the omission of separate food groups (15, 27, 28). The intake of SFA, dietary fibers and bioactive compounds is largely dependent on food choices within a given food group. However, only a small proportion of people seek the advice from an expert dietitian about proper replacement of omitted foods when transitioning to a new dietary pattern. The aim of this study was therefore to compare diet quality and serum biomarkers in healthy normal weight adults with constant body mass, who followed either LCHF, or vegan, vegetarian or omnivorous diets for at least six months, without supervision of a dietitian.

## 2. Materials and methods

### 2.1. Study design and subjects

The present study is a cross-sectional study that took place at the Faculty of Health Sciences, University of Primorska in Izola from December 2019 to October 2021. The study protocol was approved by the Slovenian National Medical Ethics Committee (No. 0120–557/2017/4) and was registered at ClinicalTrials.gov (NCT04347213).

**Figure 1:**
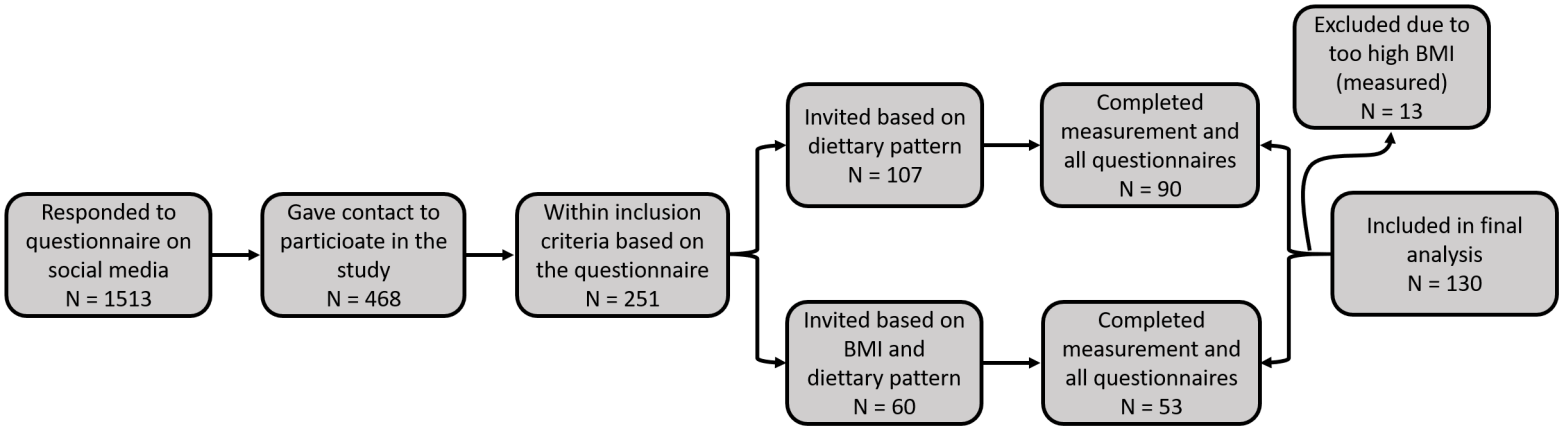
Flow diagram of the recruitment process.

Volunteers, highly interested in healthy nutrition, were recruited through a web survey posted on social media in groups dedicated to nutrition or specific dietary pattern. The survey inquired on dietary pattern, motives for its selection, duration of adherence to present dietary pattern, and self-reported body height and body mass, stability of body mass in the last three months, age, presence of chronic diseases, presence of medication, pregnancy or lactation and an invitation to give a contact for a potential invitation to participate in the study. The including criteria were adherence to dietary pattern (LCHF, vegan, vegetarian or omnivorous) for a minimum of six months, BMI between 18.5 and 30 kg/m^2^ and age between 20 and 60 years. Any chronic disease, taking medications (except contraception), being pregnant or lactating and a change in body mass (more than 3 kg) three months prior to the measurement served as the exclusion criteria. Using G*Power 3.1.9.7 (Heinrich-Heine-Univesität Düsseldorf, Germany) it was *a priori* calculated, that we need sample size N = 76 for parameter comparison between four equal groups and sample size *N* = 86 for Pearson correlation, for statistical power 0.8 at 5% 1. type error and 20% 2. type error. After reaching 90 participants, new recruits were accepted based on their sex and BMI to obtain homogenous groups (Figure 1).

Interested volunteers with no exclusion criteria received all the instructions regarding the measurements and questionnaires through an online session. Measurements included anthropometric and blood pressure measurements, blood withdrawal for biochemical analysis, 3-day Food Diary, Food Frequency Questionnaire (FFQ), Lifestyle questionnaire, International Physical Activity Questionnaire (IPAQ) and Socio-Economic Questionnaire.

### 2.2. Dietary assessment

Subjects completed 3-day Food Diary during the week before the visit at the University. They were instructed to record food intake for three days, two weekdays and one weekend day, to weigh and record all foods and beverages immediately before eating and to weigh any leftovers. They were asked to include food labels and recipes for mixed dishes and in addition, to report taking any food supplements, including the dose, and to describe if they pursue any type of fasting.

Participants also completed FFQ validated for Slovene population (29). FFQ includes nine food groups: milk and dairy products, vegetables, fruits, starchy foods, legumes, meat and meat products, fat and fatty foods, sugar and beverages. It consists of eight frequency measures: never, once per month, 2 to 3 times per month, 1 to 2 times per week, 3 to 4 times per week, 5 to 6 times per week, 1 to 2 times per day and 3 or more times per day; and 3 portion sizes: small, medium and large. Participants received visualization tools for more accurate portion assessment.

All 3-day Food Diaries and FFQ were checked by dietitian and any ambiguities and inaccuracies were addressed on the day of the measurements, to ensure the reliability of the data. Dietary data from Food Diaries and FFQ were analyzed with the Open Platform for Clinical Nutrition (OPEN).^1^ Data of macronutrient intake, micronutrient intake, energy intake, and energy density were obtained from these analyses.

#### 2.2.1. Healthy eating index

Healthy Eating Index 2015 [HEI (30)] was calculated from food diary data to evaluate diet quality according to the developers’ protocol (31). HEI evaluates intake of 13 food groups: total fruits, whole fruits, total vegetables, greens and beans, whole grains, dairy, total protein foods, seafood and plant protein foods, fatty acids ratio (sum of polyunsaturated (PUFA) and monounsaturated fatty acids (MUFA)/SFA), refined grains, sodium, added sugars and SFA, all estimated per energy intake unit (1,000 kcal). The first nine categories are scored positively and the last four are scored negatively. The sum of all categories is HEI (0–100), with the higher score representing higher diet quality.

#### 2.2.2. Dietary inflammatory index

Dietary Inflammatory Index (DII) was calculated according to author’s description (32), from 3-day Food Diaries and included 36 food parameters out of 45 (alcohol, vitamin B6, β-carotene, caffeine, dietary fibers, folic acid, garlic, ginger, magnesium, MUFA, niacin, omega-3 and omega-6 fatty acids, onion, PUFA, riboflavin, selenium, thiamin, turmeric, vitamin A, vitamin C, vitamin D, vitamin E, zinc, green/black tea, pepper, thyme/oregano, and rosemary and pro-inflammatory parameters were vitamin B12, carbohydrate, dietary cholesterol, energy, total fat, iron, protein, and SFA). Intake of every food parameter was used to calculate *z*-score based on world mean consumption, obtained from 11 datasets (32). *Z*-score was converted in percentile and centered on zero by multiplying with 2 and subtracting 1. The result was then multiplied with the overall inflammatory effect score, reported by the authors based on review of 1943 articles (32). Overall inflammatory effect scores smaller than zero were considered anti-inflammatory and scores greater than zero pro-inflammatory. Subjects’ DII score is the sum of food parameter specific DII scores. Higher DII scores represent more pro-inflammatory diets.

#### 2.2.3. Processed foods index

To assess the overall use of processed and highly processed foods in different dietary patterns, we established a new Processed Foods Index (PFI), based on the NOVA classification of processed foods (33), which serves as an indication of the degree of processed food intake:

$$PFI=\overset{m}{\underset{n=1}{\sum }}\frac{E_{food_{n}}}{EI}\mathrm{food}\;\mathrm{group}$$

where $E_{food_{n}}$ is energy value of food item n, EI is daily energy intake, foodgroup is the number of processed food group the food item belongs to and m is the number of food items consumed.

NOVA classification of processed foods classifies foods into four groups based on the processing degree: unprocessed or minimally processed foods, processed culinary ingredients, processed foods and ultra-processed foods. Group 1 includes unprocessed foods such as fresh fruits, vegetable, seeds, fungi and algae and animal foods such as muscle, offal, eggs and milk. Minimally processed foods include foods that undergo processes including drying, crushing, grinding, powdering, fractioning, filtering, roasting, boiling, non-alcoholic fermentation, pasteurization, chilling, freezing, placing in containers and vacuum packaging. Group 2 includes processed culinary ingredients such as oils, butter, lard, sugar and salt. Allowed processes in this group are pressing, refining, grinding, milling and drying. Group 3 includes canned and bottled vegetables or legumes preserved in brine, whole fruit preserved in syrup, tinned fish preserved in oil; some types of processed animal foods such as ham, bacon, pastrami, and smoked fish, freshly baked breads, and simple cheeses to which salt is added. Processes for group 3 consist of adding salt, oil, sugar or other substances from group 2 to group 1 foods and also cooking, baking and non-alcoholic fermentation. Group 4 foods are created by series of industrial techniques and processes, including carbonated soft drinks, sweets, fatty or salty packaged snacks, candies, mass produced packaged breads, buns, cookies, pastries, cakes, margarine and other spreads, sweetened breakfast cereals, fruit yoghurt, energy drinks, pre-prepared meat, cheese, pasta and pizza dishes, poultry and fish nuggets, sausages, burgers, hot dogs, powdered and packaged soups, noodles and desserts (33).

#### 2.2.4. Ketogenic ratio determination

To determine a possible effect of dietary pattern on metabolic milieu, we calculated ketogenic ratio (KR) (34, 35) with the following equation:

$$KR=\frac{0.9\;F+0.46\;P}{C+0.58\;P+0.1\;F}$$

where F is fat intake [g], P is protein intake [g] and C is carbohydrate intake [g]. KR = 1.5 was considered threshold of ketogenesis (35, 36).

### 2.3. Anthropometric measurements

Anthropometric measurements were performed after an at least 12 h overnight fast in standardized conditions with light clothing, without shoes and by the same examiner. Body height, waist and hip circumference were measured. Body mass was measured using bioelectric impedance analyzer Tanita BC 418MA (Tanita Corporation, Arlington Heights, IL, USA). Body fat percentage, fat mass, lean mass, muscle mass, total body water and phase angle were measured using bioelectrical impedance analyzer Bodystat Quadscan 4000 (Bodystat Ltd., Isle of Man, British Isles). Blood pressure was measured with an automatic blood pressure monitor Model SEM-1 (Omron Healthcare Company, Singapore).

### 2.4. Serum biomarkers

Blood samples were collected in morning hours after an overnight fast (at least 12 h). Samples were set to clot at room temperature for 30–60 min and then centrifuged at 2000 rpm for 10 min at room temperature. Serum was aliquoted and stored at −80°C until further analysis. Cobas c111 analyser (Roche, Basel, Switzerland) with a specific Cobas c111 reagent for each parameter (Roche, Basel, Switzerland) was used to determine serum glucose, triacylglycerols, total cholesterol (TC), low-density lipoprotein cholesterol (LDLC), high-density lipoprotein cholesterol (HDLC) and C-reactive protein (CRP). Non-HDL cholesterol was calculated as c(TC) – c(HDLC).

### 2.5. Gene expression analysis

To control for endogenous cholesterol synthesis, we determined the expression of HMG-CoA synthase and HMG-CoA reductase in leukocytes of participants. For the isolation of RNA from peripheral lymphocytes, blood was collected into EDTA-vacutainers (BD, Franklin Lakes, NJ, USA). Mononuclear cells were isolated from 3 ml of full blood using Histopaque-1077 (Sigma-Aldrich, St.Louis, MO, USA). Buffy coat was washed in PBS and dissolved in TriZol reagent (ThermoFisher Scientific, Waltham, MA, USA); total RNA was isolated following the manufacturers’ protocol. One μg of RNA was reverse transcribed with High-capacity cDNA Archive kit (Applied biosystems, Foster city, CA, USA). RT-PCR reaction was performed with Quant studio 3 Real-Time PCR System (Thermo Fisher Scientific, Waltham, MA, USA) and SYBR-green reagent (Qiagen, Hilden, Germany) under the following reaction conditions: 2 min at 95°C and 40 cycles of 5 s at 95°C and 10 s at 60°C. The primer sequences 196049379c2 for 3-hydroxy-3-methylglutaryl–CoA reductase (HMG-CoA-R) and 148298676c2 for 3-hydroxy-3-methylglutaryl–CoA synthase 1 (HMG-CoA-S) were selected from Primerbank (Spandidos, 2010) and 18S rRNA was used as internal control (F-GTAACCCGTTGAACCCCATT and R-CCATCCAATCGGTAGTAGCG). Primer specificity was confirmed by melting curve inspection and relative gene expressions were calculated using Δct method.

### 2.6. Statistical analysis

Statistical analysis was performed using IBM SPSS Statistics 26.0 (IBM, NY, USA). Means and standard deviations, minimum and maximum were calculated. Shapiro–Wilk test was used to evaluate the normality of data distribution. ANOVA was used to compare groups for normally distributed data and Kruskall-Wallis’ test was used for non-normally distributed data. As age has an impact on observed biochemical parameters, ANCOVA with age as a covariate was performed to compare biochemical data among the groups. Hierarchical regression analysis was performed to examine the effects of age, nutritional parameters known to affect serum cholesterol levels and dietary indices on TC. *p*-values < 0.05 were considered statistically significant.

## 3. Results

### 3.1. Subjects characteristics

**Figure 2:**
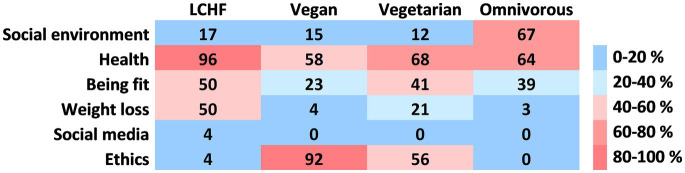
Motives for choosing the current dietary pattern. More than one motive could have been chosen by each participant. Results are shown as percentage of each group.

Two hundred thirty seven individuals, identifying themselves as practicing LCHF diet, replied to our online survey, 98 of whom gave their contact for a potential participation in the study. After screening for inclusion criteria, we were able to recruit 24 adults with 3-months stable body mass, practicing LCHF diet for at least six months (58% from 1–3 years and 25% more than 3 years). They mostly reported choosing their diet for health (Figure 2). For comparison, we recruited 32 vegans, 37 vegetarians and 37 omnivores, the sample thus included a total of 130 subjects (97 females and 33 males) (Table 1). The subjects were healthy adults without any chronic disease, and with comparable BMI and other anthropometric parameters. A total of 76% of subjects were in relationship or married and 29% had high school diploma, 58% bachelor’s degree and 13% master’s degree or PhD. More than half (62%) of the subjects never smoked, 21% were former smokers and 17% were current smokers (regular or occasional).

**Table 1:** Characteristics of study subject by dietary pattern.

Dietary pattern	LCHF	Vegan	Vegetarian	Omnivorous	All
N (M/F)	24 (5/19)	32 (9/23)	37 (7/30)	37 (12/25)	130 (33/97)
Age (years)*	41.2 ± 5.7^a,b,c^	34.0 ± 10.1	37.4 ± 10.7	36.2 ± 11.5	36.9 ± 10.2
Height (cm)	170 ± 9	170 ± 9	169 ± 8	171 ± 9	170 ± 9
Body mass (kg)	67.9 ± 13.0	62.9 ± 8.0	64.6 ± 10.0	66.2 ± 13.3	65.2 ± 11.2
BMI (kg/m^2^)	23.4 ± 3.1	21.8 ± 2.1	22.4 ± 2.6	22.6 ± 3.0	22.5 ± 2.8
Fat mass (%)	23.9 ± 6.0	20.8 ± 8.4	24.3 ± 7.1	21.9 ± 7.1	22.7 ± 7.3
Fat free mass (kg)	51.3 ± 11.4	49.8 ± 8.9	48.9 ± 9.3	51.9 ± 12.8	50.4 ± 10.6
TBW (%)	56.2 ± 4.8	57.8 ± 7.2	55.2 ± 5.8	57.0 ± 5.5	56.6 ± 6.0
SBP (mmHg)	121 ± 9	123 ± 16	121 ± 16	119 ± 12	121 ± 14
WHR	0.79 ± 0.05	0.79 ± 0.06	0.80 ± 0.05	0.79 ± 0.06	0.79 ± 0.06
WHtR	0.45 ± 0.04	0.44 ± 0.05	0.45 ± 0.04	0.45 ± 0.06	0.45 ± 0.05
PA (MET)	7.7 ± 6.1	11.4 ± 11.5	10.2 ± 10.3	13.4 ± 10.0	10.9 ± 10.0
Smoking (N (%))	6 (25)	4 (13)	6 (16)	6 (16)	22 (17)

LCHF, low carbohydrate high fat diet; BMI, Body Mass Index; WHT, Waist to Hip Ratio; WHtR, Waist to Height Ratio; TBW, Total body water; SBP, Systolic blood pressure; PA, Physical activity. *Kruskal-Wallis H test, *p* < 0.05; Student’s or Mann–Whitney test was performed for all the parameters expressed as mean ± standard deviation between all pairs of groups. Statistically significant differences (*p* < 0.05) are reported with superscript indices close to the first value of the pair: ^a^LCHF and vegan group; ^b^LCHF and vegetarian group; ^c^LCHF and omnivorous group.

### 3.2. Nutritional intake

**Figure 3:**
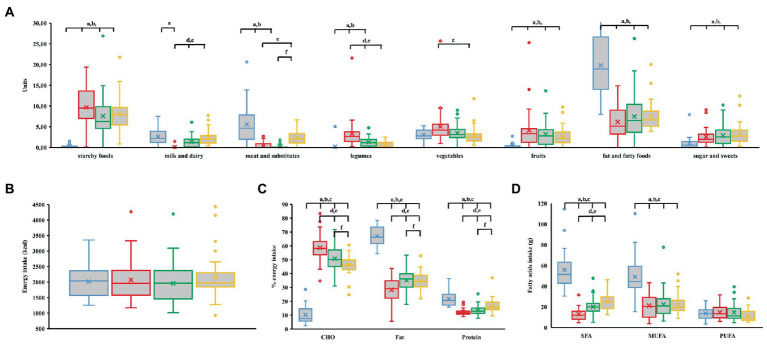
**(A)** Food group unit intake (meat and substitutes include vegan meat substitutes). **(B)** Daily energy intake. **(C)** Contribution of macronutrients to daily energy intake. **(D)** Fatty acids intake. LCHF, low carbohydrate high fat; CHO, carbohydrate; SFA, saturated fatty acids; MUFA, monounsaturated fatty acids; PUFA, polyunsaturated fatty acids. Blue—LCHF, red—Vegan, green—Vegetarian, yellow—Omnivorous. ^index^*p* < 0.05, Student’s or Mann–Whitney test between: ^a^LCHF and vegan group; ^b^LCHF and vegetarian group; ^c^LCHF and omnivorous group; ^d^vegan and vegetarian group; ^e^vegan and omnivorous group; ^f^vegetarian and omnivorous group.

Energy intake did not differ among the groups (*p* = 0.607; Figure 3B). However, there were significant differences in the intake of food groups (Figure 3A). LCHF had significantly higher intake of fats and fatty foods, and lower intakes of starchy foods, fruits, sugars and sweets than all other groups. Additionally, it differed from vegan group in the intake of milk and dairy products and from both vegan and vegetarian group in the intake of legumes and meat and substitutes. LCHF group had significantly different macronutrient distribution than other groups (*p* < 0.001; Figure 3C): it had the lowest intakes of carbohydrates and the highest intakes of fats and proteins. All participants from LCHF group met the reference values of at least 0.8 g protein per kg body mass per day (11) (Table 2). The minimal recommended protein intake was not reached in 31% vegans, 35% vegetarians and 8% omnivores. For the intake of carbohydrates, the recommended 50% EI (11) was achieved in 84% vegans, 54% vegetarians and 22% omnivores. Fat intake below the recommended maximum of 30% EI (11) was determined in 53% vegan, 22% vegetarian and 19% omnivorous participants. LCHF group had significantly higher SFA and MUFA intake than other groups (Figure 3D).

**Table 2:** Percentage of participants meeting reference value.

Nutrient	RV	LCHF % meet RV	Vegan % meet RV	Vegetarian % meet RV	Omnivorous % meet RV
Carbohydrate	>50% EI^1^	0	84.4	54.1	21.6
Fat	<30% EI^1^	0	53.1	21.6	18.9
Protein	>0.8 g/kg BM^1^	100	68.8	64.9	91.9
Free sugar 1	<10% EI^2^	95.8	93.8	86.5	81.1
Free sugar 2	<5% EI^2^	95.8	68.8	40.5	35.1
Dietary fibers	>30 g^1^	8.3	71.9	43.2	35.1
ω-3/ω-6	>0.20^1^	45.8	40.6	43.2	54.1

BM, body mass; EI, energy intake; LCHF, low carbohydrate high fat; RV, reference value. ^1^Slovenian RV (11), ^2^WHO RV (37).

LCHF group had the lowest number of meals per day and the lowest eating time frame (Table 3). LCHF group had the highest intake of animal proteins while vegans had the highest intake of plant proteins. LCHF group had the lowest sugar and free sugar intake (Table 3). For this parameter, the highest intake was determined in omnivorous group, but also here it did not exceed reference values (RV) in 81% of the group. LCHF group had the lowest dietary fibers intake (*p* < 0.001; Table 3), but a marked proportion of individuals in other three groups also did not meet the RV (Table 2).

**Table 3:** Energy and macronutrient intake.

Variable (Unit)	LCHF	Vegan	Vegetarian	Omnivorous
Meals (N/day)*	2.8 ± 0.5 ^a,b,c^	3.8 ± 0.7	3.7 ± 0.9 ^f^	4.1 ± 0.8
Eating time frame (h/day)*	8.7 ± 1.9 ^a,b,c^	11.3 ± 1.4 ^e^	11.0 ± 1.8 ^f^	12.0 ± 1.6
Energy density (kcal/g)	1.22 ± 0.44	1.12 ± 0.29	1.15 ± 0.36	1.07 ± 0.33
Ketogenic ratio*	1.50 ± 0.36 ^a,b,c^	0.27 ± 0.10	0.35 ± 0.10	0.38 ± 0.10
Carbohydrate (g)*	51 ± 35 ^a,b,c^	307 ± 123 ^d,e^	252 ± 106	254 ± 107
Sugar (g)*	28.3 ± 22.6 ^a,b,c^	90.4 ± 58.7	85.9 ± 42.4	90.7 ± 42.6
Free sugar (g)*	9.2 ± 14.4 ^a,b,c^	22.0 ± 19.2 ^d,e^	30.9 ± 21.8	36.6 ± 24.8
Free sugar (%)*	1.7 ± 2.3 ^a,b,c^	4.3 ± 3.2 ^e^	6.1 ± 3.5	6.7 ± 3.9
Fat (g)*	150 ± 46 ^a,b,c^	64 ± 27	76 ± 27	81 ± 20
SFA (% EI)*	25.0 ± 6.0 ^a,b,c^	5.8 ± 2.9 ^d,e^	9.6 ± 3.7	10.8 ± 2.7
MUFA (% EI)*	21.9 ± 6.1 ^a,b,c^	9.4 ± 4.7	10.3 ± 4.6	9.6 ± 3.4
PUFA (% EI)*	6.1 ± 2.2	6.5 ± 2.5 ^e^	6.8 ± 2.9 ^f^	4.8 ± 2.2
ω-6 FA (% EI)*	5.0 ± 1.9 ^a,b,c^	3.1 ± 2.2	3.6 ± 2.4	2.9 ± 1.9
ω-3 FA (% EI)*	0.9 ± 0.5 ^a,b,c^	0.7 ± 0.6	0.7 ± 0.8	0.6 ± 0.5
ω-3/ω-6 FA ratio	0.24 ± 0.15	0.35 ± 0.60	0.23 ± 0.20	0.27 ± 0.21
Cholesterol (mg)*	1,078 ± 500 ^a,b,c^	12 ± 16 ^d,e^	162 ± 140 ^f^	314 ± 166
Protein (g)*	108 ± 35 ^a,b,c^	65 ± 31^e^	65 ± 23 ^f^	91 ± 42
Protein (g/kg BM)*^1^	1.60 ± 0.45 ^a,b,c^	1.02 ± 0.36 ^e^	1.02 ± 0.41 ^f^	1.40 ± 0.61
Animal protein (%)*^2^	86.0 ± 12.8 ^a,b,c^	1.7 ± 4.8 ^d,e^	29.8 ± 20.2 ^f^	59.9 ± 15.1
Dietary fibers (g)*	17.9 ± 21.1 ^a,b,c^	50.4 ± 42.7 ^d,e^	31.7 ± 14.9	27.1 ± 15.3
Alcohol (g)*	4.9 ± 9.1	2.7 ± 7.5 ^e^	2.1 ± 4.9 ^f^	6.6 ± 10.6

All values are expressed as mean ± standard deviation. LCHF, low carbohydrate high fat; FA, fatty acids; SFA, saturated FA; MUFA, monounsaturated FA; PUFA, polyunsaturated FA; EI, energy intake; BM, body mass. *p < 0.05, ANOVA or Kruskal-Wallis H test; Student’s or Mann–Whitney test was performed for all the parameters between all pairs of groups. Statistically significant differences (*p* < 0.05) are reported with superscript indices close to the first value of the pair: ^a^LCHF and vegan group; ^b^LCHF and vegetarian group; ^c^LCHF and omnivorous group; ^d^vegan and vegetarian group; ^e^vegan and omnivorous group; ^f^vegetarian and omnivorous group. ^1^Recommended values for protein intake is 0.8 g/kg BM (11). ^2^Percent of protein intake (animal protein + plant protein = 100%).

**Figure 4:**
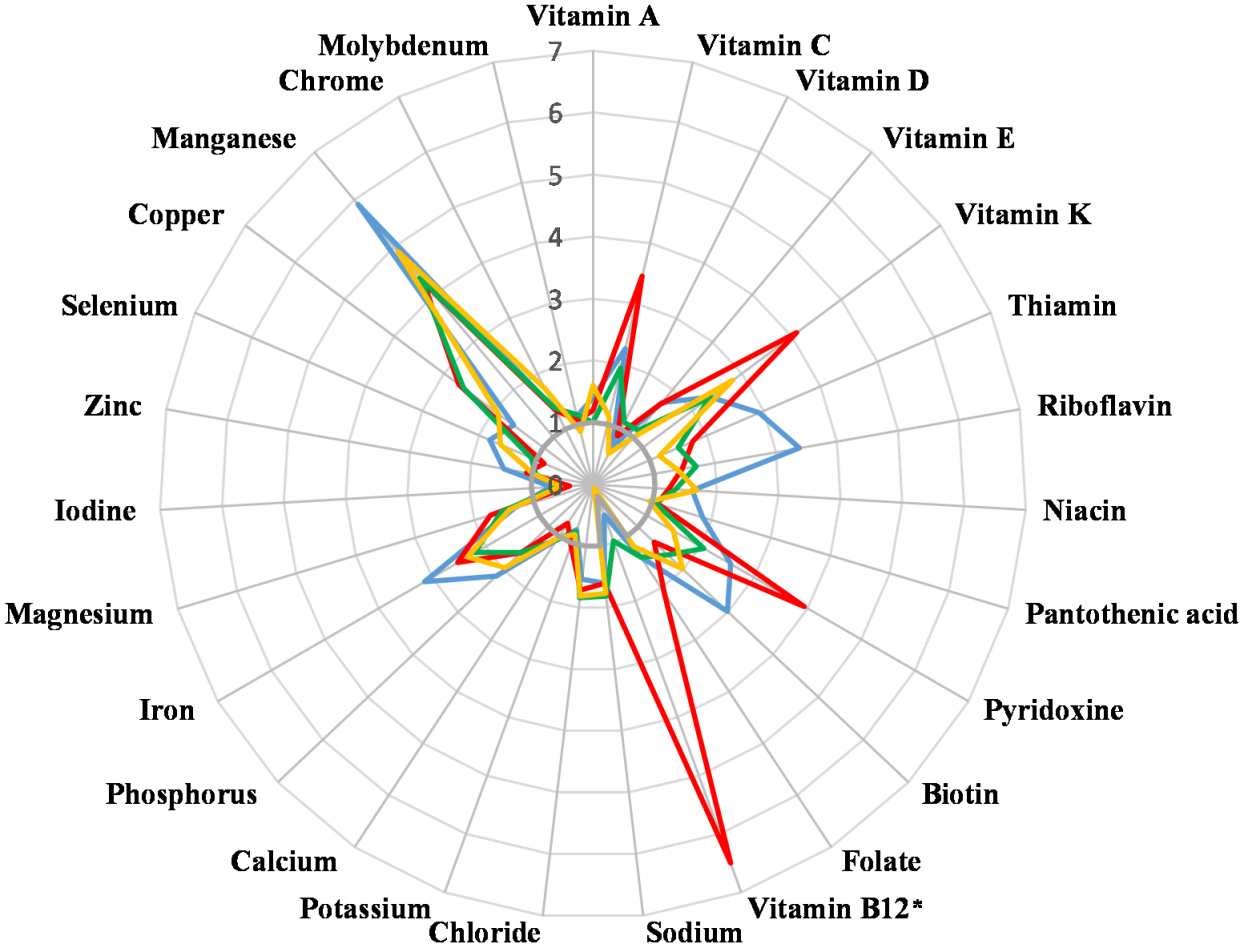
Micronutrient intake in multiples of Slovene Reference Values [SRV, (11)]. *Vitamin B_12_ values were divided by 20 to fit in the chart. Blue—Low carbohydrate high fat, red—Vegan, green—Vegetarian, yellow—Omnivorous.

In LCHF group, analysis of micronutrient intakes revealed significantly lower intake of copper and significantly higher intake of vitamin A, phosphorus and manganese than in vegan and vegetarian group, significantly higher intake of vitamin C and pyridoxine than in omnivorous group, and higher intake of vitamin E, riboflavin, pantothenic acid, biotin, zinc and selenium than in all other groups (Figure 4). For iodine, only LCHF group reached RV (11). Vegans had the lowest calcium intake, below RV. Omnivores had vitamin B_12_ intakes around RV, whereas the other groups exceeded RV by multiples of tens. The majority of participants on LCHF and vegan diet have taken at least one dietary supplement per day (75 and 84%, respectively), while this percentage was lower among vegetarians (46%) and omnivores (43%). The three most common dietary supplements used were vitamin B_12_, vitamin D and vitamin C. All the supplements were included in the analysis of micronutrient intakes (Figure 4).

#### 3.2.1. Dietary indices

In addition to the analysis of macro- and micronutrient intakes, three dietary parameters, HEI, DII and PFI, were calculated to evaluate the quality of the diet (Table 4). LCHF had the lowest diet quality as determined by HEI, followed by omnivores, vegetarians and vegans. Average HEI for all dietary groups together was 65.6 ± 13.5. DII calculated from the total intakes of foods and dietary supplements was significantly different between groups. The lowest DII, which indicates the lowest intake of potentially inflammatory foods, was observed in vegan group. PFI did not significantly differ between groups. HEI negatively correlated with both DII and PFI, while DII and PFI positively correlated.

**Table 4:** Dietary indices.

Dietary index	LCHF	Vegan	Vegetarian	Omnivorous
HEI*	51.1 ± 8.1 ^a,b,c^	73.4 ± 11.9 ^d,e^	68.2 ± 10.9	65.5 ± 13.1
Total fruits*	0.83 ± 0.96 ^a,b,c^	3.84 ± 1.44	3.30 ± 1.85	3.30 ± 1.33
Whole fruits*	1.29 ± 1.63 ^a,b,c^	4.12 ± 1.52	3.68 ± 2.02	4.03 ± 1.48
Total vegetables*	3.63 ± 1.35 ^c^	4.06 ± 1.01 ^e^	3.68 ± 1.23 ^f^	2.95 ± 1.13
Greens and beans*	1.33 ± 1.76 ^a,b,c^	4.31 ± 1.20 ^d,e^	3.14 ± 1.72	2.68 ± 1.75
Whole grains*	0.37 ± 1.28 ^a,b,c^	6.47 ± 3.95	5.73 ± 3.91	5.62 ± 3.93
Dairy*	6.62 ± 2.96 ^a,b^	0.38 ± 1.13 ^d,e^	4.30 ± 3.53 ^f^	6.14 ± 2.72
Total protein food*	4.92 ± 0.41 ^a,b,c^	3.53 ± 1.52	3.27 ± 1.48 ^f^	4.00 ± 1.08
Seafood and plant proteins*	3.21 ± 2.13 ^a^	4.50 ± 1.34 ^e^	4.22 ± 1.40	3.78 ± 1.69
Fatty acids*	1.54 ± 1.96 ^a,b^	8.19 ± 2.92 ^d,e^	5.16 ± 3.94 ^f^	2.32 ± 3.14
Refined grains*	10.00 ± 0.00 ^a,b,c^	7.94 ± 3.38	8.49 ± 2.78	8.11 ± 2.68
Sodium	7.37 ± 3.26	7.03 ± 3.96	7.19 ± 3.49	7.35 ± 3.20
Added sugar*	9.92 ± 0.41 ^b,c^	9.66 ± 0.97 ^d,e^	9.30 ± 1.13	9.00 ± 1.45
Saturated fats*	0.08 ± 0.41 ^a,b,c^	9.41 ± 1.90 ^d,e^	6.81 ± 3.17	6.27 ± 2.91
DII*	1.85 ± 1.45 ^a^	1.02 ± 1.75 ^d,e^	1.89 ± 1.79	2.43 ± 2.39
PFI	2.23 ± 0.29	2.26 ± 0.42	2.42 ± 0.44	2.42 ± 0.39
Ketogenic ratio	1.50 ± 0.36 ^a,b,c^	0.27 ± 0.10 ^d,e^	0.35 ± 0.11	0.38 ± 0.10

All values are expressed as mean ± standard deviation. LCHF, Low carbohydrate high fat; HEI, Healthy Eating Index; DII, Dietary Inflammatory Index; PFI, Processed Food Index. *ANOVA or Kruskal-Wallis H test, *p* < 0.05; Student’s or Mann–Whitney test was performed for all the parameters between all pairs of groups. Statistically significant differences (*p* < 0.05) are reported with superscript indices close to the first value of the pair: ^a^LCHF and vegan group; ^b^LCHF and vegetarian group; ^c^LCHF and omnivorous group; ^d^vegan and vegetarian group; ^e^vegan and omnivorous group; ^f^vegetarian and omnivorous group.

### 3.3. Serum biomarkers

To analyze whether the different nutrient intakes reflect in serum biochemical parameters, lipid profile, glucose and CRP were measured. Among the participants in the LCHF group, 71% had TC above the reference value and 67% had increased LDLC level (Table 5). As the age of participants in the LCHF group was higher compared to other groups and due to the known association of TC with age, ANCOVA model with the age as a covariate was additionally performed. With this covariate considered, the levels of TC [*F* (3, 125) = 11.23; *p* < 0.001], LDLC [*F* (3, 125) = 10.41; *p* < 0.001] and non-HDLC [*F* (3, 125) = 7.89; *p* < 0.001], but also HDLC [*F* (3, 125) = 8.43; *p* < 0.001] were still significantly different among groups. TC and LDLC levels were the lowest in vegan group, where the levels were significantly lower also when compared to the omnivorous group not only to LCHF. The same was observed for HDLC level. In fact, 19% of vegan participants had LDLC levels below the reference value. For HDLC, the percentage of participants with too low levels was similar for vegan, vegetarian and omnivorous (22, 19, 19%, respectively), whereas in the LCHF group it was only 4%. In the levels of glucose, triacylglycerols and CRP there were no important differences between groups, also, all the levels were within reference values.

**Table 5:** Serum biomarkers.

Biomarker (Unit)	LCHF		Vegan		Vegetarian		Omnivorous		RV
Glucose (mmol/L)	4.64 ± 0.60		4.71 ± 0.44		4.67 ± 0.54		4.79 ± 0.46		3.6–6.1
	^<^0%	^>^0%	^<^0%	^>^0%	^<^0%	^>^0%	^<^0%	^>^0%	
Total cholesterol (mmol/L)*	7.48 ± 4.27^a,b,c^		4.01 ± 0.86^d,e^		4.46 ± 0.90		4.84 ± 1.84		4.0–5.2
	^<^8%	^>^71%	^<^50%	^>^6%	^<^41%	^>^19%	^<^22%	^>^22%	
HDLC (mmol/L)*	2.10 ± 0.47 ^a,b^		1.58 ± 0.41^e^		1.72 ± 0.42		1.87 ± 0.47		>1.4
	^<^4%		^<^22%		^<^19%		^<^19%		
LDLC (mmol/L)*	5.85 ± 4.47 ^a,b,c^		2.66 ± 0.76^e^		3.00 ± 0.89		3.14 ± 1.30		2.0–3.3
	^<^0%	^>^67%	^<^19%	^>^16%	^<^8%	^>^35%	^<^11%	^>^30%	
Non-HDLC (mmol/L)*	5.38 ± 4.26 ^a,b,c^		2.42 ± 0.91 ^e^		2.74 ± 0.93		2.98 ± 1.90		<3.4^1^
		^>^59%		^>^9%		^>^19%		^>^22%	
TAG (mmol/L)	0.91 ± 0.66		0.88 ± 0.39		0.91 ± 0.39		1.05 ± 1.02		0.6–1.7
	^<^25%	^>^8%	^<^13%	^>^3%	^<^16%	^>^5%	^<^24%	^>^11%	
TAG/HDL	0,466 ± 0,357		0,672 ± 0,628		0,584 ± 0,362		0,663 ± 0,856		♂ < 2.967^2^
		^>^0%		^>^3%		^>^0%		^>^5%	♀ < 2.237^2^
CRP (mg/L)	0.61 ± 0.50		0.80 ± 1.08		0.75 ± 1.01		1.20 ± 1.77^#^		<2.0^3^
		^>^4%		^>^6%		^>^3%		^>^19%	

All values are expressed as mean ± standard deviation. LCHF, Low carbohydrate high fat; CRP, C-reactive protein; HDLC, high density lipoprotein cholesterol; LDLC, low density lipoprotein cholesterol; non-HDLC = TC - HDLC; TAG, triacylglycerols; RV, reference values (unless a reference is indicated, Slovene reference values are reported); ^<^% of participants with levels below recommended values; ^>^% of participants with levels above recommended values; ^#^value for 36 participants, one was excluded due to very high CRP values of 15.61 mg/L, indicating an acute infection (CRP values indicating chronic low-grade inflammation are between 2 and 10 mg/L (38); *ANOVA or Kruskal-Wallis H test controlled for age, *p* < 0.05. Student’s or Mann–Whitney test was performed for all the parameters between all pairs of groups. Statistically significant differences (*p* < 0.05) are reported with superscript indices close to the first value of the pair: ^a^LCHF and vegan group; ^b^LCHF and vegetarian group; ^c^LCHF and omnivorous group; ^d^vegan and vegetarian group; ^e^vegan and omnivorous group; ^f^vegetarian and omnivorous group. ^1^Reference (39), ^2^Reference (40), ^3^Reference (38).

Further, hierarchical regression analysis was performed to examine the effects of age, nutritional parameters known to affect serum cholesterol levels and dietary indices on the total serum cholesterol levels (Table 6). In the first step, age was entered, followed by specific nutrients intake (SFA, MUFA, PUFA, dietary cholesterol, animal protein, and dietary fibers) in the second step. In the third step, dietary indices were entered (HEI, DII and PFI). Stage one (age) explained 9.7% of the variation of TC [*F* (1, 128) = 13.709; *p* < 0.001]. Stage two (SFA, MUFA, PUFA, cholesterol, dietary fibers and animal protein intakes) explained additional 40.0% of the variation of TC [*F* (7, 122) = 17.211, *p* < 0.001]. And at stage three, the proposed regression model explained 51.4% of total variance in cholesterol levels [*F* (10, 119) = 12.608; *p* < 0.001]. Of the chosen independent variables, age and daily intakes of SFA and MUFA proved to significantly impact TC level (Table 6).

**Table 6:** Hierarchical regression model for total serum cholesterol.

Predictor	Dependent variable: total serum cholesterol			
	ΔR^2^	*β*	*F*	*p*
Step 1	0.097		13.709	0.000
Age (years)		0.311		0.000
Step 2	0.400		17.211	0.000
Age (years)		0.204		0.003
SFA intake (g)		0.644		0.000
MUFA intake (g)		−0.273		0.016
PUFA intake (g)		−0.081		0.353
Cholesterol intake (mg)		0.234		0.065
Dietary fibers (g)		−0.020		0.792
Animal protein (g)		−0.066		0.603
Step 3	0.018		12.608	0.000
Age (years)		0.176		0.012
SFA intake (g)		0.713		0.000
MUFA intake (g)		−0.292		0.010
PUFA intake (g)		−0.106		0.259
Cholesterol intake (mg)		0.165		0.218
Dietary fibers (g)		−0.071		0.369
Animal protein (g)		−0.071		0.575
HEI		0.032		0.727
DII		0.001		0.994
PFI		−0.145		0.070
Model	0.514		12.608	

SFA, Saturated fatty acids; MUFA, Monounsaturated fatty acids; PUFA, Polyunsaturated fatty acids; HEI, Healthy Eating Index; DII, Dietary Inflammatory Index; PFI, Processed Food Index.

For some insight whether the observed differences in the cholesterol levels might be due to different endogenous synthesis, we further analyzed the expression of HMG-CoA synthase and HMG-CoA reductase in peripheral lymphocytes. The analysis was performed on a subsample (*N* = 88), where 22 participants were randomly selected from each group. No statistically significant difference was found among groups (respectively *p* = 0.742 and *p* = 0.945), controlling for age.

**Figure 5:**
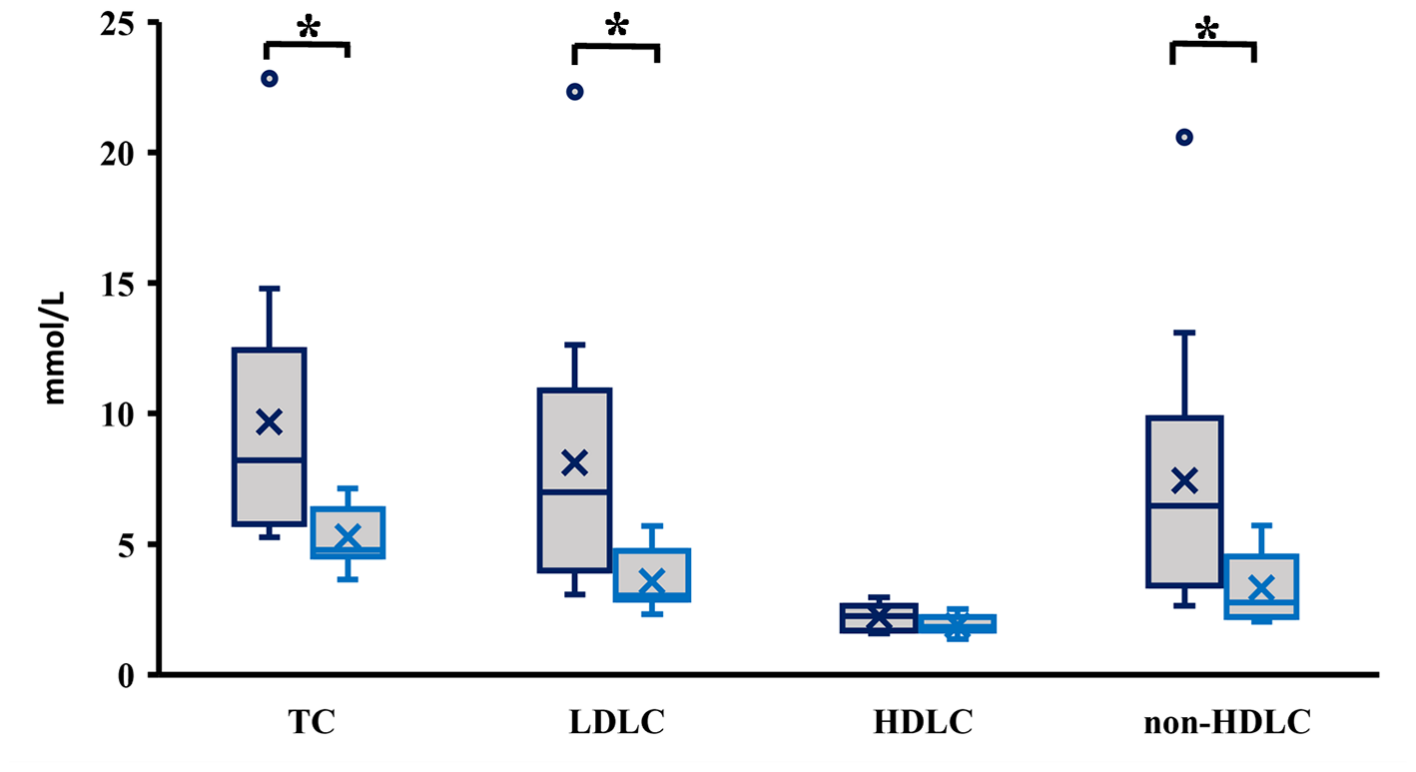
Cholesterol levels in low carbohydrate high fat group divided based on ketogenic ratio (KR). Dark blue—KR > 1.5; light blue—KR < 1.5. *p < 0.05; TC, Total Cholesterol; LDLC, Low-density lipoprotein cholesterol; HDLC, High-density lipoprotein cholesterol; non-HDLC = TC – HDLC.

The calculated ketogenic ratio was in LCHF group from 0.74 to 2.10. The participants were therefore on both sides of the threshold of ketogenesis, which is set at KR = 1.5 (35, 36). When we divided LCHF group into two subgroups, those below the threshold of ketogenesis (*N* = 12) and those above (*N* = 12), subjects above the threshold had significantly higher levels of TC, LDL and non-HDL (*p* = 0.008, *p* = 0.009 and *p* = 0.014, respectively; Figure 5).

## 4. Discussion

LCHF is advertised as long-term safe either for improving health or for improving sport performance, but in scientific literature some concerns are raised. To assess the quality and health effects of long-term self-planned LCHF diet in healthy lean adults we compared it to the most commonly followed omnivorous diet and to two patterns with whole food group omission – vegan and vegetarian. We have focused only on the participants who had stable body mass for at least three months; as LCHF is often used as a weight loss program, potential nutrient deficiencies may be due to the restriction of energy intake and not only arising from omitting whole food groups, but on the other hand, energy restriction may also mask some negative effects of high fat intakes. Although studies report lower BMI in vegetarians compared to non-vegetarians and even lower in vegans (16, 41), in order to objectively compare the four dietary patterns, we recruited groups of participants with comparable BMI, as BMI is an independent risk factor for low grade inflammation, metabolic syndrome, type 2 diabetes mellitus and cardiovascular diseases (42).

### 4.1. Macronutrient intakes

By definition, LCHF diet differs from other diets in the intake of carbohydrates and fats. Consistent with LCHF definition (7, 10), the intake of carbohydrates in our participants was up to 26% EI and was significantly lower than in other groups. A complete omission of starchy foods, legumes and fruits was observed. Some of the LCHF participants followed a ketogenic diet, while the rest lowered their carbohydrate intake without an interest in being ketotic, which was manifested in a wide range of KR. The observed variety of low-carbohydrate dietary patterns is likely a consequence of diverse lay guidelines and publications, available online and advertised by various nutritional coaches and was therefore expected in self-advised planning. Increased fat intake in LCHF group was mostly achieved with milk and dairy, meat and substitutes and fat and fatty food intake, which reflected in higher intakes of SFA, MUFA and cholesterol, while PUFA intake in LCHF did not differ from other groups. LCHF group had also the highest protein intake, which was again achieved through the intake of animal protein. In contrast, this group had the lowest intake of plant proteins among the groups. Of the three control groups, vegan had the highest intake of carbohydrates and the lowest intakes of fats and proteins.

We have analyzed to what extent the other dietary patterns were in line with the national recommendations (11). In vegan group, the highest proportion of participants met the RV for the intake of carbohydrate and fat. Similar to what was reported on a national level (6), our participants exhibited a tendency towards lower carbohydrate intake and higher fat intake, not just those following LCHF but also others. The majority of participants reached the RV for proteins [0.8 g/kg BM day (11)], however the percentage was somewhat lower in vegan and vegetarian group (69 and 65%, respectively, compared to 100% in LCHF and 92% in omnivorous). Among vegans, 31% did not reach the RV, which is additionally concerning because plant-derived proteins have lower absorbability and may have lower content of essential amino acids; therefore higher intakes of plant proteins are recommended (43). All groups had low intake of free sugars with the majority of participants meeting the WHO recommendations (37). LCHF group had low dietary fibers intake; only 8% met the RV and the average intake was 17.9 g per day. In other groups the intakes were better but still low; two thirds of omnivores, more than half of the vegetarians and one third of vegan did not meet the RV. However, when we consider the average intake of dietary fibers in each group, the participants in the three control groups had higher dietary fibers intakes than previously reported for Slovenian adults. There, nearly 90% of adults did not meet the RV (44).

### 4.2. Micronutrients intakes

Despite the fears of micronutrient insufficiencies (14, 15) due to omissions of starchy foods, legumes and fruits, mean values of micronutrient intake in LCHF group met or even exceeded recommended intakes for the majority of micronutrients, but mostly remained under the upper tolerable limits, where established. The result can be partially explained by the fact that 75% of the participants from this group regularly took at least one dietary supplement. In addition, in comparison to some previous reports (15), where low micronutrient intakes were reported, participants in the present study had no EI restriction. Nevertheless, we observed too low intakes of potassium, iodine and vitamin D, while mean value of calcium intake coincided with RV, indicating a presence of insufficient intakes in a part of participants. Inadequate micronutrient intakes were noted also in other groups. Intakes of vitamin D were insufficient in all groups which is in line with a previous report on a national level (45); despite the endogenous biosynthesis of vitamin D, the majority of Slovene population has insufficient serum 25(OH)D levels, especially in winter. Iodine intakes were also too low in all groups. The main source of iodine in Slovenia is iodized salt and iodine sufficiency in Slovenia is reportedly achieved due to highly excessive salt intake (46). Our participants did not exceed salt intake recommendations to such a high degree as reported for general population in Slovenia (47). Further, non-iodized salt is believed to be more natural and healthy in some laic nutritional information sources preferred by people on special dietary patterns, especially vegetarian and vegan, and is available on market (48), which is of concern. Similar to LCHF group, calcium intake was problematic in part of vegetarians and omnivores, and majority of vegans. Other insufficient intakes were group specific: vegans did not meet the RV for selenium; vegetarians did not meet RV for potassium and zinc; while omnivores had too low intakes of vitamin E, pantothenic acid, zinc and molybdenum. The use of supplements was common also in these groups, as 84% of vegan, 46% of vegetarians and 43% of omnivorous participants took at least one dietary supplement per day. The results suggest that the participants following restrictive dietary patterns are familiar with potential insufficiencies and try to correct them with supplements. However, none of the self-advised diets was fully sufficient in providing all nutrients, thus further education of the public is necessary.

### 4.3. Dietary quality assessed through dietary indices

Assessing a diet on the level of singular nutrients does not give the exact picture of the possible impact of the ingested food mixtures on health. An overall assessment of diet quality gives further information (49, 50) and different indices have been developed for this purpose. We have chosen three: HEI, which assesses overall diet quality and was associated with better cardiovascular markers, overall health and lower mortality rates (49), DII that measures inflammatory potential of the diet and has been associated with cardiovascular diseases incidence and related mortality (51) and PFI, a novel index which was based on NOVA classification of processed foods (33), since the consumption of ultra-processed food was also associated with adverse health effects (52). LCHF group had significantly lower HEI than other groups. Their score was low for whole grains, which was expected in line with LCHF definition; fatty acids ratio and SFA intake, which could be improved with a better choice of fatty foods; and was the best of all the groups for refined grains and free sugars intake, again in line with the definition. LCHF had a better score in total vegetables than omnivorous and the best score of all the groups for total protein food. Omnivorous had very similarly low score for fatty acids ratio, but higher for SFA than LCHF group. The highest HEI was observed in vegans, followed by comparable results in vegetarians and omnivorous group. We point out that the lowest HEI observed in LCHF group was still relatively high as it was comparable to HEI observed for overall population in several European countries (50). DII of LCHF group was comparable to that of vegetarians and omnivores, while vegans had significantly lower DII, which points to the consumption of less pro-inflammatory food constituents (32) in this group. As DII is a population-based index, it is difficult to compare our results to the results reported in literature. There was no difference in PFI among our groups. In all groups it was between 2 and 3, which suggests a low presence of ultra-processed foods in their diets.

### 4.4. Serum biomarkers

Serum biomarkers were assessed to observe the health footprint of the observed diets. Relatively high diet quality and a comparable low consumption of pro-inflammatory foods in all groups reflected in low mean CRP levels of all groups [much lower than 2 mg/L which is a well-accepted cut-off level indicating chronic low-grade inflammation (38)], without statistical difference among the groups. The biggest part of participants exceeding the cut-off of chronic low-grade inflammation was in omnivorous group. All the participants had glucose levels within the recommended values and there were no differences among the groups. Mean triacylglycerols levels were within the recommended values in all groups and there were no differences among the groups. LCHF group had significantly higher cholesterols levels. Seventy-one percent of participants in LCHF group had TC levels above the upper limit, many had also too high LDLC (67%) and non-HDLC (59%) – the latter is recommended as a cardiovascular disease prediction factor by European Society of Cardiology (53). The literature suggests low carbohydrate diets might have an impact on LDL particle size, increasing LDL peak, but not mean, particle size and decreasing the numbers of small dense LDL and total LDL particles, which may reflect a decreased atherogenicity of the LDL particles, but the clinical significance of LDL particles is still unknown (54). TAG/HDLC ratio (40) and non-HDLC [in individuals without hypertriacyglycerolaemia (55)] have been suggested as potential biomarkers of small dense LDL particles with high specificity and sensitivity determined by ROC curves. Our LCHF participants had significantly higher non-LDLC, but did not differ from other groups in TAG/HDLC ratio. For a final conclusion LDL subfraction analysis would have to be performed. Previously, we and others have not observed an increase in cholesterols levels in shorter weight-loss ketogenic diet interventions in participants with obesity (22, 24, 56). Ketosis induces a different metabolic milieu than diets where the presence of carbohydrate and/or protein is sufficient to elicit an insulin response and KR was suggested as suitable to determine the metabolic effect of a diet (35). We therefore divided the participants of the LCHF group according to their KR to those with higher probability of being in ketosis (KR > 1.5) and those with lower. The participants with KR > 1.5 had a worse cholesterol profile. Compared to the aforementioned ketogenic interventions with positive results on cholesterol profile that were hypocaloric and where the participants had regular meetings with dietitians (22, 24, 56), participants in this study had stable body mass, an eucaloric diet and did not consult a dietitian. Research suggests that lowering carbohydrate intake and subsequent lowering of insulin levels may inhibit hepatic cholesterol synthesis which might result in a lower TC and LDLC as long as one does not increase SFA and dietary cholesterol intakes while lowering carbohydrate intake (20). Significant correlations between SFA intake and TC, LDLC, HDLC and non-HDLC have been observed (39, 57). To control for endogenous cholesterol synthesis, we determined the expression of HMG-CoA synthase and HMG-CoA reductase in leucocytes of participants and observed no differences among groups. Even though liver expressions should be analyses for a final answer, this points to exogenous factors to be the explanation for the observed cholesterol profile.

Cholesterol has a fundamental role in cellular membrane in all cell types, in steroid hormones and bile acid production, which raises a concern that very low cholesterol levels observed in some participants, mostly vegan and vegetarian, might also have a negative impact on health. Very low LDLC and HDLC levels were associated with increased risk for stroke, cataract and all-cause mortality (58, 59). Some of our participants had LDLC (19% vegans, 8% vegetarians and 11% omnivorous) and HDLC (22% vegans and 19% vegetarians and omnivorous) levels below RV. Of note, the reported level of LDLC that increased the risk for all-cause mortality [<1.55 mmol/L (59)] is lower than Slovene recommended minimum.

### 4.5. Nutritional choices that might explain the observed cholesterol profile in LCHF participants

Our present LCHF participants had higher absolute SFA intake than those reported in the aforementioned ketogenic interventions (22, 24, 56). Although our participants were motivated for LCHF diet for health-related reasons, it seems that their only focus was lowering the carbohydrate intake without paying much attention to the quality of other macronutrients and diet quality in general. Mediterranean ketogenic diet low in SFA intake and high in MUFA and PUFA intake was reported to maintain normal cholesterol levels (20, 60). In agreement, our linear regression model identified SFA intake as a positive predictor for total serum cholesterol and MUFA intake as a negative predictor. A higher emphasis on selection of healthy fat sources from vegetables, high in MUFA and PUFA, or fish, high in omega-3 PUFA, when substituting carbohydrate as source of energy with fat in LCHF diet should be made in lay literature.

Apart from SFA intake, high dietary fibers intake is demonstrated to have a positive effect on cholesterol profile (61). High viscosity dietary fibers trap and eliminate bile, consequently lowering TC and LDLC without affecting HDLC (62). Although the present regression model did not reveal dietary fibers intake as a significant predictor of TC, LCHF group had low intakes of all dietary-fibers-rich foods such as whole grains, fruits and vegetables. Dietary fibers intake below recommendations was observed also in omnivores, who had low intakes of vegetable and whole grains and in fact, they had significantly higher TC levels than vegetarian and vegan group, although those were within reference values. In public, carbohydrates are often blamed for obesity, high serum glucose and development of chronic noncommunicable diseases (63), but omitting complex carbohydrates has a negative effect on dietary fibers intake, which was previously associated with increased risks for noncommunicable diseases (64). Since carbohydrate intake is limited in LCHF diet, RV for dietary fibers intake is hard to reach without inclusion of processed foods in this group. We should point out, that some processed foods are healthy and have the potential to lower serum cholesterol levels. This is true for processed extra virgin olive oil and canola oil, processed foods with added plant sterols or stanols, and foods with added soluble dietary fibers and some fermented foods (65); all of these food groups produced at least a moderate (i.e., 0.20–0.40 mmol/L) reduction in LDL cholesterol levels and are suitable for a LCHF diet. Functional foods enriched with dietary fibers content such as inulin, galactooligosaccharides, fructooligosaccharides and lactulose are good examples of foods for increasing the dietary fibers content without increasing digestible carbohydrate intake (66) and are again suitable for LCHF diet. Functional foods with added inulin were associated with improved serum lipid and glucose profile (67). Different dietary fibers supplements are appearing on the market, also the ones containing high viscous dietary fiber such as β-glucan, psyllium, and raw guar gum, and have been shown to lower LDLC (62).

Another possible explanation for different effects of LCHF diets on cholesterol profile lies in the fact that controlled studies of ketogenic diet with favorable lipid profile results had a controlled protein intake (22, 24, 56). High protein intake in the present LCHF group could influence metabolic regulation and cause a shift in macronutrient consumption for energy source. Further, animal protein sources contributed mainly to this high protein intake. Although our regression model did not reveal animal protein intake as significant predictor of TC, increased animal protein intake was previously associated with decreased longevity (68), contrary to the intake of plant proteins which was associated with decreased risk for cardiovascular and all-cause mortality (69). Similar conclusion was drawn from cohort studies investigating low-carbohydrate diet. There, low-carbohydrate diet with predominant animal food sources was associated with higher all-cause, cardiovascular and cancer mortality, while low-carbohydrate diet with predominant plant food sources was associated with lower all-cause and cardiovascular mortality (70, 71). In this context it is important that plant protein intake is accompanied with higher intake of phytochemicals and dietary fibers (72), while animal protein sources contain a lot of SFA. The public, interested in LCHF diet, should therefore be encouraged to substitute some of their animal protein sources with plant protein sources.

### 4.6. Limitations

The relatively small sample size in the present study may be considered a limitation, as we were able to recruit 24 participants practicing LCHF diet with stable body mass and suitable BMI. The prevalence of people who follow LCHF diet in Slovenia is not known, but national dietary study Si.Menu 2017/18, performed using EFSA methodology on a representative sample of 364 Slovenian adults (18–64 years old), did not detect people on LCHF diet (6). The fact that we were only recruiting participants following the same pattern for a minimum of six months while keeping stable body mass, strongly limited our sample size. The target sample size for vegan and vegetarian participants was easier to reach, as these patterns are more frequent in Slovenian population; according to the national survey approximately 1.6% of population does not consume meat (6). Although participants in all groups fell within the same age-range, participants in LCHF group were statistically older. This might have an impact on cholesterol levels. Indeed, age explained 10% of TC variability in our model, but an additional 40% of TC variability was explained by SFA and MUFA intakes.

## 5. Conclusion

Most of our vegan and vegetarian participants have chosen their diet for health reasons and to be fit, but many did so also for ethical reasons. In contrast, LCHF pattern was chosen exclusively to improve or maintain health. Furthermore, all of our participant were individuals with an above average interest in nutrition. This reflected in a higher HEI than reported for European adults (50). In spite of that fact, self-planned LCHF diet showed poor nutritional choices – in particular by simply substituting carbohydrate-derived energy with fat without selecting the fat source. Literature suggests a restriction or elimination of consumption of processed and unprocessed red meat, starchy vegetables and refined grains and an emphasis on dietary fibers derived from whole grains, dietary-fibers-rich fruit, low-carbohydrate vegetables (cruciferous and green leafy vegetables and legumes), avocado, olive and vegetable oils, soy, fish and chicken to constitute a healthy LCHF diet (23). Low dietary fiber intakes could be mitigated also with functional foods with added dietary fibers or with dietary fiber supplements, especially the highly viscous ones. Further effort on educating the public about healthy low-carbohydrate choices but also correct replacements of omitted foods in vegan and vegetarian patterns should be made to achieve healthier diets in “real life” while respecting individuals’ decision on which dietary pattern they wish to follow.

## Data availability statement

The raw data supporting the conclusions of this article will be made available by the authors, without undue reservation.

## Ethics statement

The studies involving human participants were reviewed and approved by Republic of Slovenia Ministry of Health National Medical Ethics Committee (No. 0120-557/2017/4). The patients/participants provided their written informed consent to participate in this study.

## Author contributions

NM and ZJ: conceptualization, supervision, and project administration. NB, KS, SK, AP, ZJ, and NM: methodology and investigation. NM and NB: formal analysis and data curation. ZJ: resources. NB: writing—original draft preparation. NM, ZJ, SK, and AP: writing—review and editing. NM: visualization. All authors contributed to the article and approved the submitted version.

## Funding

The study was supported by the Slovenian Research Agency (Programs P1-0386 and I0-0035).

## Conflict of interest

The authors declare that the research was conducted in the absence of any commercial or financial relationships that could be construed as a potential conflict of interest.

## Publisher’s note

All claims expressed in this article are solely those of the authors and do not necessarily represent those of their affiliated organizations, or those of the publisher, the editors and the reviewers. Any product that may be evaluated in this article, or claim that may be made by its manufacturer, is not guaranteed or endorsed by the publisher.

## Footnotes

^1^http://opkp.si/
